# Supplementary material for: Diagnostic Peptide Discovery: Prioritization of Pathogen Diagnostic Markers Using Multiple Features
Source: PLoS One. 2012 Dec 14;7(12):e50748. doi: 10.1371/journal.pone.0050748 (PMC3522711; doi:10.1371/journal.pone.0050748)
Supplement: Materials S1 — Selected peptide profiles. Two sets of protein peptide-score profiles, are included as supplementary materials, for the purposes of visualization of prioritized peptides. The plots are explained in Figure 1. A Peptide score profiles for the high-scoring proteins included in the arrays. Additional information on the selected peptides can be found in Table S3. B Peptide score profiles for the top 1000 protein candidates (ranked by their highest scoring peptide). For clusters of orthologous genes, only the best candidate of the cluster is considered. (ZIP) [file pone.0050748.s003.zip › Data-S1B-TcPeptideProfiles/479_Tc00.1047053508897.20.html]

|  |  |
| --- | --- |
| Rank | 479 |
| Gene Name | Tc00.1047053508897.20 |
| Gene Id | 33223 |
| Description | signal recognition particle protein, putative |
| Ortholog Group | OG1.2\_3032 |
| Length | 763 |
| Gene Copies | 1 |
| Signal Peptide | 0.998 |
| SP Cleavage | 0.998 |
| Mass Spec Density | 0 |
| Glyco Density | 0 |
| GPI Tail | 0 |
| GPI Cleavage | 0 |
| CAI | 0.489 |
| Localization Score | 0.431 |
| Expression Score | 0 |
| Protein Score | 2.155 |
| Max.Pep Score | 7.250 |
